# Surgery

**Published:** 1896-02

**Authors:** 


### SURGERY.

EDITED BY FLOYD W. MCRAE, M.D.

### ICHTHYOL FOR ERYSIPELAS.

In a long article by Eberson (*Wien Med. Presse*) bearing on ichthyol and its action, the author confirms the reports extant regarding the antiseptic, reducing, and antiphlogistic properties of this remedy, its efficacy in the treatment of various skin and women diseases. He then adds a report of a few cases that have come under his observation. He was called to see a boy suffering with facial erysipelas. For two days the author followed the usual treatment, but without success. The boy then presented the following symptoms: Temperature, 102.7° F.; nose much swollen; face red and bloated, the redness extending on both sides as far as the neck, and up as far as the scalp; headache and nausea. On the third day the author began the following treatment: Application of a 30-per-cent. solution of ichthyol in glycerin; application of ice; tampon of a 5-per-cent. ichthyol solution to the nose, and quinine internally. The next day the temperature was down to 99.5° F., and there was marked improvement every way. After three days of treatment the fever no longer existed, and the face had diminished one-half in size. Recovery thereupon quickly followed. After the first application of ichthyol the boy remarked that the tension of the skin and the pain were diminishing.

The second case reported is that of a 2½-months-old boy affected with erysipelas of the right side of the face; his temperature was 102° F. Dr. E. made an application with the ichthyol solution two days in succession; on the third day the author found that the child had recovered.

In a third instance the author treated a woman, over 70 years of age, afflicted with erysipelas of the leg, by means of ichthyol; she recovered after two days of treatment. A relapse occurred two months later, but a repetition of the treatment brought about a cure.

The author has twice had occasion to test the remedy on himself.

Dr. E. considers ichthyol a specific against erysipelas. He employs a 50-per-cent. solution in glycerin for adults, and a 35-per-cent. solution with children.

These solutions he freely applies, with a bristle brush, in concentric rings, beginning about an inch from the edge of the inflamed skin, and finally painting the center several times.—*American Medico-Surgical Bulletin.*

---

### THE RADICAL CURE OF FISTULA IN ANO BY AN IMPROVED METHOD.

Dr. Augustin H. Goelet, of New York, in the *American Medico-Surgical Bulletin,* July 1, describes an improved operation for fistula in ano which does away with the usual troublesome after-treatment in these cases by securing primary union, and affords complete retentive power, even if there are two fistulæ and the sphincters must be divided in two places. This result he obtains by carefully uniting the raw surfaces by means of sutures, introducing first buried catgut, continuous sutures for the deeper structures, including the muscles, and separate interrupted sutures of chromic gut for the mucous membrane and perineum.

He emphasizes the importance of previous divulsions of the sphincter, strict asepsis, thorough curettage of all fistulous tracks, the use of buried sutures for uniting the deeper structures and separate superficial sutures for the mucous membrane only, and inactivity of the bowels for five days following the operation.

In favor of this method of suturing as against the introduction of sutures through the mucous membrane of the rectum to include the deeper structures, the danger of infection by leakage along the track of the suture is avoided, as well as obstruction to the circulation and consequent edema which would interfere with primary union.

---

### LOCAL ANESTHESIA BY SCHLEICH'S METHOD.

By this method the anesthetic property of cocaine is reinforced by the addition of morphine, sodium chloride, and carbolic acid to the solution. Schleich uses three different solutions, called respectively strong, medium, and weak. These are:

℞ Cocain hydrochlorate..........3 grains.
Morphin hydrochlorate..........$\frac{1}{3}$ grain.
Sodium chlorid..........3 grains.
Distilled water..........3 ounces.
Mix. Sterilize and add of a 5 per cent. solution of carbolic acid, 2 drops.
Label.—Anesthetic solution No. 1.—Strong.

℞ Cocain hydrochlorate..........$1\frac{1}{2}$ grains.
Morphin hydrochlorate..........$\frac{1}{3}$ grain.
Sodium chlorid..........3 grains.
Distilled water..........3 ounces.
Mix. Sterilize and add of a 5 per cent solution of carbolic acid, 2 drops.
Label.—Anesthetic solution No. 2.—Medium.

℞ Cocain hydrochlorate..........$\frac{1}{6}$ grain.
Morphin hydrochlorate..........$\frac{1}{12}$ grain.
Sodium chlorid..........3 grains.
Distilled water..........3 ounces.
Mix. Sterilize and add of a 5 per cent. solution of carbolic acid, 2 drops.
Label.—Anesthetic solution No. 3.—Weak.

The solution is injected *into* the skin, not beneath it. The local anesthesia lasts from two to twenty minutes. The small quantity of cocaine contained in these solutions makes it impossible to use a poisonous dose. This method is said to be decidedly superior to the usual way of using cocaine hypodermically.

---

### TENDON GRAFTING—A NEW OPERATION FOR DEFORMITIES FOLLOWING INFANTILE PARALYSIS, WITH REPORT OF SUCCESSFUL CASE.

At a meeting of the New York State Medical Association, October 15, 1895 (*Med. Rec.* Oct. 26), Dr. S. E. Milliken presented a boy eleven years of age upon whom twenty months before he had successfully grafted part of the extensor tendon of the great toe into the tendon of the tibialis anticus muscle, the latter having been paralyzed since the child was eighteen months old.

The case which was presented showed the advantages of only taking part of the tendon of a healthy muscle which was made to carry on the function of its paralyzed associate, without in any way interfering with its own work.

The brace which had been worn since two years of age was left off, the patient walked without a limp, the talipes valgus was entirely corrected and the boy had become quite an expert on roller skates.

Dr. Milliken predicts a great field for tendon grafting in these otherwise hopeless cases of infantile paralysis, who heretofore have been doomed to the wearing of braces all their lives.

---

### IODOFORM OINTMENT INJECTIONS IN THE TREATMENT OF SUPPURATIVE ADENITIS OF THE GROIN.

Dr. James R. Hayden, of New York, recommends the following method, which has been employed by him in the treatment of buboes with very satisfactory results: The operative field having been shaved and rendered surgically clean, a few drops of a four per cent. solution of cocaine are injected beneath the skin where the puncture is to be made. The pus is then evacuated and thoroughly squeezed out through a small puncture. The abscess cavity is then injected with pure peroxide of hydrogen until the fluid returns practically clear. It is then washed out with a 1-5000 bichloride solution and injected with a ten per cent. iodoform ointment. Then a cold bichloride dressing is applied with the idea of congealing the ointment. The patient should be kept quiet for forty-eight hours, although it is not necessary that he be confined to bed. The dressings are removed on the third or fourth day.

---

### SURGICAL TUBERCULOSIS.

For abscesses, ulcers, and joint affections: Open freely, and excise if joint be involved. Remove the caseous material by curetting, sponge the parts well and arrest the bleeding. Fill the cavity, through a thick rubber tube, with salt solution kept at the boiling-point; fill and empty the cavity until it is thoroughly cauterized. Or, fill the cavity with a cold or lukewarm salt solution, and raise the liquid to a boiling point by introducing into it the blade of a thermo-cautery at a red heat. One minute is sufficient to heat the water in a cavity as large as a pigeon's egg. Do not touch the walls of the cavity with the blade of the knife. Give an anesthetic if necessary, though usually cocaine locally will be sufficient.—JEANNEL, *Midi Medical.*

